# Defining and detecting links in chromosomes

**DOI:** 10.1038/s41598-019-47999-4

**Published:** 2019-08-13

**Authors:** Szymon Niewieczerzal, Wanda Niemyska, Joanna I. Sulkowska

**Affiliations:** 1 Centre of New Technologies, University of Warsaw, Banacha 2c, 02-097 Warsaw, Poland; 2 Faculty of Mathematics, Informatics, and Mechanics, University of Warsaw, Banacha 2, 02-097 Warsaw, Poland; 3 Departament of Chemistry, University of Warsaw, Pasteura 1, 02-093 Warsaw, Poland

**Keywords:** Chromosomes, Computational models, Pure mathematics

## Abstract

Sophisticated methods for mapping chromatin contacts enable to generate data of the genome structure that provide deep insights into the formation of chromatin interactions within cell nuclei. Due to the recent progress in this field, three-dimensional genomic structures of individual haploid mouse embryonic stem cells have been determined. Here, we analyze these data (8 cells) and determine comprehensive landscape of entanglements between interphase chromosomes. We find a significant number of stable links formed by chromosome pairs. Some links are even conserved between cells. Moreover, examples of stable multiple links, with at least three chromosomes engaged, are also identified. Types of links and their location along chromosomes are determined based on computations of HOMFLY-PT polynomials and Gauss Linking Numbers. Furthermore, stability of links is studied between different models, cells, and based on relaxation simulations of the genomic structure in a simplified structure-based representation. Identified links suggest that small fraction of chromosomes are entangled not only locally. How topoisomerases engineer such configurations remains an open question. Furthermore, presented methods can be used as a quantitative assessment – descriptor – to distinguish the quality of modeled data.

## Introduction

In the cell cycle process, chromosomes undergo extensive structural reorganization. During mitosis, in order to successfully segregate genetic information, chromatin fibers adopt highly complex cylindrical shape. In the interphase, however, chromosomes unfold and become organized in distinct territories with particular shapes depending on the species. The process of packing the eukariotic genome in preparation for cell division is one of the most important unsolved problems in molecular biology^1–3^. Complex biomolecular machinery, with crucial role of enzymes such as condensin, cohesin and topoisomerases, is neccessary to transform the interphase genome into mitotic chromosomes. However, most of the processes involved in the structural rearrangement of the genome (into the mitotic chromosomes) still remain unclear^4–6^. The complex topology within DNA molecules arises because of being tightly crammed inside a confined environment. DNA molecules are likely to be knotted by random intersegmental passages, and the unfavorable entanglements should be removed, e.g. type II topoisomerases can change the topology of DNA strands by passing one double helix through another^7–10^.

Recent progress in determining 3D structures of individual genomes, however, can shed some light on their entanglement^6,11–16^. In particular, it was shown, that three-dimensional models of the human diploid genome remain minimally knotted^17^ or almost unknotted^18,19^. Small steady state fractions of DNA knots have been found common in intracellular chromatin^20^. The analysis of individual mammalian genomes in^21^ has shown that apart from the unmappable regions of the genome, the structure is well determined, and 80% of chromosome chains contain knots^22^.

High packing of multiple DNA molecules within eukaryotic cell nuclei could lead to the existence of entanglements between two different chromosomal chains. Although chromosomes in the interphase occupy well defined territories, they are positioned close to each other, which gives an opportunity for mixing or interchromosomal passages. Therefore a natural question arises − are there links in chromosomes?

The entanglement between interphase chromosomes has not been analyzed, to the best of our knowledge. The aim of this work is to investigate to what extent densely packed interphase chromosomes in an eukaryotic nucleus are entangled, and to determine types and location of links. Here, we use the experimental data provided in^21^ obtained by calculating contact matrices by application of the Hi-C method^23^ to individual genomes of haploid embryonic mouse stem cells. These contacts were next used as constraints in simulated annealing of chromosome chains, treated as beads connected by strings, in order to determine the 3D structure of the genome (details are provided in the supporting information of^21^).

**Figure 1:**
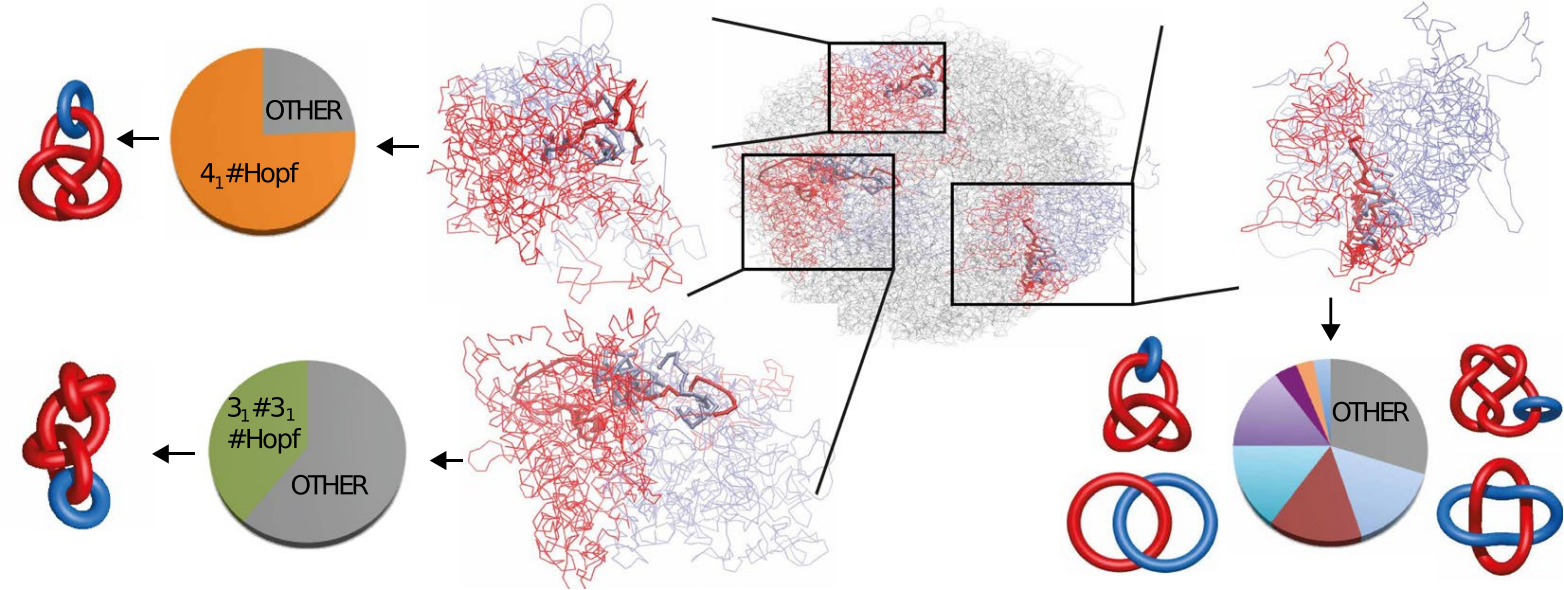
Example of results for the genome from cell no. 5^21^ for three selected chromosome pairs: *d*−*h*, *l*−*n*, and *r*−*t*. High values of the linking numbers indicate presence of the links. For each of these pairs, exact topology of the link is further determined by computing the HOMFLY-PT polynomial for 100 random closures. Obtained probabilities are presented in a form of a circle chart. For both pairs on the left, there is one dominating topology. “Other” in the chart denotes highly complex links with more than 9 crossings. The most probable links for these pairs are shown in a schematic representation next to probability charts. For the chromosome pair presented on the right, there are several topologies with significant probabilities.

As our goal is to comprehensively analyze entanglement between chromosome pairs (but not only), we combine two methods. One of them is based on calculations of HOMFLY-PT polynomials^24,25^. In contrast to Alexander polynomial, which is extensively used in KnotProt server^26^ to analyze entanglement in proteins, HOMFLY-PT polynomial can distinguish splittable (unlinked) links, and much more complex links. The second is based on calculations of Gauss Linking Number (GLN) given by the Gaussian integrals^27^. This method provides an alternative measure of mutual linking of two loops. An illustrative example of an application of both methods (approaches) is presented in Fig. 1. Here, we show that in the analyzed data 8% of chromosome pairs are linked. Furthermore, we investigate conservation of links in all available experimental models and their robustness by means of short molecular dynamics simulations of the genomic structure in a simplified structure-based representation. An alternating link may indicate inaccurately predicted fragment of the genome in the vicinity of the indicated link. Next we analyze conservation of links (preserved in previous methods) between cells. Finally, we show that some chromosomes form stable multicomponent links.

## Materials and Methods

### Links

**Figure 2:**
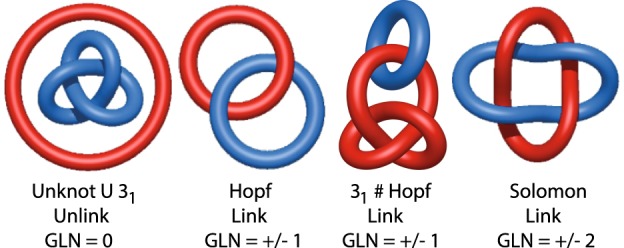
Examples of two component links and their GLN values. The GLN describes how many times one chain (chromosome) winds around another one. The symbol # denotes composite links, i.e. sums of prime knots (e.g. 3_1_) and links. The symbol U denotes two unlinked chains.

Links in three dimensional Euclidean space can be described as a system of nonintersecting closed curves (which interchangeably we call components, chains or loops). A link with one component is called a knot. In this article we study mainly links with two components, where each component is an individual chromosome. Examples of links are shown in Fig. 2. The fact that chromosomes from eukaryotic cells are open chains is a significant complication, since components of mathematical links should form closed loops. Thus it is a challenge to describe entanglement of chromosomes using classical mathematical tools. To achieve this goal we combine two methods: calculating HOMFLY-PT polynomial and Gauss Linking Number (GLN). HOMFLY-PT polynomial identifies some specific types of links, while GLN only detects the existence and a ‘strength’ of a link, counting how many times one chain (chromosome) winds around another one. However, to compute HOMFLY-PT polynomial, closed loops are needed, thus we need to close the chromosome chains, which is not a unique operation and can lead to different links. On the other hand, GLN can be calculated for open chains, and its computation is much faster. Both methods are described in more detail below.

### HOMFLY-PT polynomial

To identify a type of a link we compute its HOMFLY-PT polynomial^24,25^ (after closing all loops, as we describe below). The core of our code is based on the Ewing-Millet implementation^28^ and it was used before to identify knotted^26,29^ and linked^30^ proteins. Generally, the HOMFLY-PT polynomial is harder to compute than the Alexander polynomial, but it distinguishes between a greater number of knots and links, including composite structures (see Fig. 2), splittable links, and links with different chiralities and orientations. This is crucial for chromosomes, since they, contrary to proteins^31^, often form very complex, composite links, as we show in the Results section. Our software can distinguish between all splittable links up to 9 crossings, all prime links up to 8 crossings, all composite links consisting of the Hopf link up to 7 crossings, and some other more complicated links that occur in pairs of chromosomes (altogether 200 links). If the link has more crossings we call it *Other* and in such case we do not know if it is splittable (i.e. if some of its components are unlinked). In such situations GLN method is especially helpful.

Before computing HOMFLY-PT polynomials, we simplify structures of chromosomes - in particular reducing some of their residues and thus making them shorter - using the Koniaris-Muthukumar-Taylor (KMT)^32^ algorithm, following the procedure described in^33^.

### Closure methods

**Figure 3:**
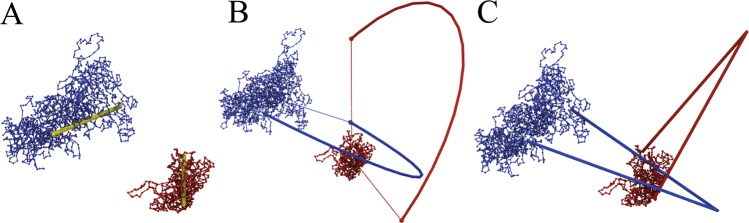
Various methods of connecting chromosome endpoints, based on cell 2 and a pair of chromosomes *d* and *r*. Panel A - direct closure method, identified link: 3_1_ U 3_1_. Panel B - the center of mass method, identified link: 3_1_ # 3_1_ # Hopf. Panel C - random closure method, identified links: 3_1_ U 3_1_ (68%), *Other* (29%), 3_1_ # 3_1_ # *Hopf* (3%). The GLN value between chromosomes is −0.009, indicating unlinked topology in agreement with the direct closure (**A**) and the random closure (**C**) methods. Note that in the center of mass method (**B**) chromosomes are closed by additional arcs, which in this particular case form an (artificial) link.

The HOMFLY-PT polynomial can be calculated only for closed loops, and therefore chromosome chains need to be closed first. One may consider three different approaches to closing an open chain: (i) direct closure method, in which chromosome endpoints are connected by the shortest interval; (ii) the center of mass method, in which endpoints are connected to two points on a large sphere along the direction of a line connecting the center of mass of the chromosome and the respective endpoints; those two points are next connected by an arc on that sphere to close the chain; and (iii) random closure method, in which for each chromosome we choose randomly one point on a huge sphere and connect both chromosome endpoints with it by direct segments; we repeat that procedure several times, each time checking the link type of obtained closed structure, and finally we find out which link type occurs most often. One has to keep in mind that each closure method can introduce additional artificial entanglements, in an individual chromosome or between a choromosome pair. For the graphical illustration of different closure methods see Fig. 3.

In our studies of chromosome pairs we used the random closure method, taking into account the results of 100 random closures each time. The center of mass method is applied typically when investigating the knot type of a single chromosome. Whenever some other method is used, we mention it explicitly.

### Gauss linking number (GLN)

The linking number (GLN) between two closed curves *γ*_1_ and *γ*_2_ is given by the Gaussian double integral

$$GLN\equiv \frac{1}{4\pi }{\oint }_{{\gamma }_{1}}{\oint }_{{\gamma }_{2}}\frac{{\overrightarrow{r}}^{\mathrm{(1)}}-{\overrightarrow{r}}^{\mathrm{(2)}}}{|{\overrightarrow{r}}^{\mathrm{(1)}}-{\overrightarrow{r}}^{\mathrm{(2)}}{|}^{3}}\cdot (d{\overrightarrow{r}}^{\mathrm{(1)}}\times d{\overrightarrow{r}}^{\mathrm{(2)}}),$$

where ${\overrightarrow{r}}^{\mathrm{(1)}}$ and ${\overrightarrow{r}}^{\mathrm{(2)}}$ parametrize the two curves. Gauss proved that for closed curves this integral is always integer, it is an invariant up to isotopies, and indicates how many times one curve winds around the second one. However the integral can be calculated for open chains too, such as eukaryotic chromosomes. Even though its value is then no longer integer, it still indicates the character of linking, i.e. the value of GLN close to ±1 means that two chains form a structure analogous to the Hopf link (however its components can be themselves knotted, see Fig. 2), and the value close to ±2 suggests a presence of a link analogous to the Solomon link. The GLN detects the orientation of chains and can be positive or negative, and its high absolute values |*GLN*| indicate linking. In this paper we often consider only such an absolute value to detect linking of two chromosomes.

We stress that the GLN provides an information only about linking of two chromosomes, but it does not detect topological details of each of them. In particular the second and the third case in Fig. 2 have the same linking number, and thus the GLN method cannot distinguish them.

Previously we used the GLN to study new entangled motifs in proteins called lassos^34^. Lassos occur in structures with disulfide (or other) bridges, where at least one terminus of a protein backbone pierces through a covalent loop (closed by such a bridge). In^35^ we found that if |*GLN*| between two fragments of a protein chain is higher than 0.6, then there is a high probability that they are linked (specifically: in 93% cases the lasso loop is pierced by the tail if |*GLN*| between them is higher than 0.6). In this paper we do not define the threshold value of |*GLN*| that would indicate a presence of a link. However, sometimes in our analysis we focus on pairs of chromosomes with |*GLN*| higher than 0.7 or higher than 1.0.

Moreover, one may use the GLN method to find out the exact location of the linking between chains. It can be accomplished by calculating GLN values between fragments of both chains (which is done in the most part while calculating final GLN value between whole chains and thus it does not introduce significant additional computational costs) and choosing those fragments which are the shortest but GLN value between them still remains similar to the one between whole chains.

### Relaxation procedure

Molecular dynamics simulations of the 3D genome were introduced to assess the significance of entanglements present in the experimentally derived nuclear chromosomal structures. The structure based representation of the system, in which the provided structure minimizes the potential function, is of the following form:

1 $$\begin{array}{rcl}V & = & \sum _{bonds}\,{k}_{b}{(r-{r}_{0})}^{2}+\sum _{angles}\,{k}_{a}{(cos(\theta )-cos({\theta }_{0}))}^{2}\\  &  & +\,\sum _{dihedrals}\,[{k}_{d}^{1}\mathrm{(1}+cos(\varphi -{\varphi }_{0}))+{k}_{d}^{3}\mathrm{(1}+cos\mathrm{(3(}\varphi -{\varphi }_{0})))]\\  &  & +\,\sum _{contacts}\,4\alpha \varepsilon [{(\frac{\sigma }{r})}^{12}-{(\frac{\sigma }{r})}^{6}]+\sum _{non-contacts}\,4\varepsilon {(\frac{\sigma }{r})}^{12},\end{array}$$

with the following force constants: *k*_*b*_ = 20000.0 *ε*/*nm*^2^, *k*_*α*_ = 20.0 *ε*, ${k}_{d}^{\mathrm{(1)}}=1.0\,\varepsilon$, ${k}_{d}^{\mathrm{(3)}}=0.5\,\varepsilon$, *α* = 0.2. Two beads are considered to form a contact if they do not interact along the chain, and they fulfil the distance condition in the initial structure: their separation is not larger than the cut-off distance *r*_*coff*_ = 2.0 nm, and not shorter than *r*_*min*_. Pairs of nonbonded beads which in the initial structure are within *r*_*min*_ distance do not interact with each other (there is no penalty for overlapping). Otherwise the repulsive interaction is applied. If a bead is within a distance of *r*_*min*_ = 0.6 nm of the preceding bead along the chain, it is removed. Reduced temperature *T* is definied by $\tilde{T}={k}_{B}T/\varepsilon {\tilde{k}}_{B}$, where ${\tilde{k}}_{B}=0.00831451$. Each genomic system was subject to 200,000 time steps with the time step *dt* = 0.0005. All molecular dynamics simulations were conducted using GROMACS^36^.

### Visualisation

Structures of chromosomes were visualised using VMD^37^, and schematic link structures were generated using KnotPlot program^38^ and homemade scripts by P. Dabrowski-Tumanski.

## Results

### Links in genomic structure

The experimental data provided by Stevens *et al*.^21^ contains contact maps calculated by means of Hi-C method for eight individual genomes of mouse haploid stem cells. For each of these cells, ten 3D coarse-grained structure models of the genome were generated, using procedure based on simulated annealing (as described in detail in the supporting information of^21^). Individual genome contained 20 chromosomes comprising between 582 and 1925 beads each (one bead represents approximately 100 kb).

First, we calculated occurrences of links in all models for each cell (for details see table SI in the supplementary material). There are $$(\begin{array}{c}20\\ 2\end{array})=190$$ chromosome pairs in each cell’s genome. The number of linked pairs varied between 7 for cell no. 2 and 28 for cell no. 3, calculated as an average over the models. On the other hand, the number of links in the models representing the same cell was very similar. This is why for simplicity in the following analysis we considered the model no. 1 as a representative one for each cell.

**Figure 4:**
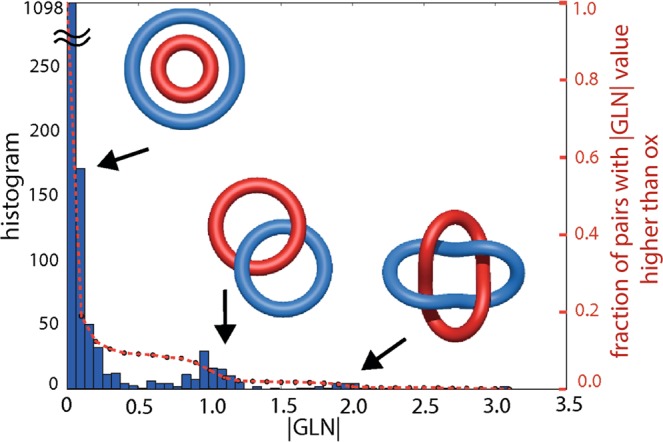
Blue: the number of chromosome pairs as a function of |*GLN*| value calculated for all 8 cells. For each cell, model no. 1 was considered. Red: cumulative probability for this distribution.

In these calculations we considered that two chromosome chains form a link if their |*GLN*| ≥ 0.7. The |*GLN*| values calculated for all cells (each represented by model no. 1) are shown as a histogram in Fig. 4. They are not integers since eukaryotic chromosomes are open chains (in such case the linking number returns non-integer values). However, |*GLN*| calculated for a pair of chromosomes is generally close to the ideal (integer) value characteristic for the given link (0 for unlink, 1 for Hopf link, 2 for Solomon link, etc.).

About 80% of pairs have |*GLN*| close to zero, which means that there is no link formed between these chromosomes. For another 10% of pairs, |*GLN*| is still lower than 0.5, thus we expect that they also do not form links. Finally, as much as 126 chromosome pairs, which is roughly 8%, form links, having |*GLN*| greater than 0.7. Most of them, about 100, are expected to form links of Hopf type, in Fig. 4 represented by the distinct peak at |*GLN*| around 1. There are also 20 pairs which, as we suspect, can form Solomon type link, and another 6 that can be even more entagled.

Some of these links might be artificial, as a result of measuring equipment resolution and then experimental data processing; but still, some chromosomes in the interphase genome may be entangled in a highly complex way.

### Verification of the closure methods

To detect a link between chromosome chains we calculate the |*GLN*| value, however this method does not provide the full information about the type of a link. To determine the link type, we calculate in addition HOMFLY-PT polynomials. Contrary to |*GLN*|, this method requires analyzed chains to form closed curves. A construction of such closures is a sensitive step, as it is in general not unique and can introduce additional crossings, which change the link type.

In the analysis of links in chromosomes we compared three closure methods. The first one is the direct closure method, in which endpoints of link components are connected by a straight segment. In this method closing of a chromosome can alter its link type (knot types of chromosomes forming the link, in particular) when its ends are on the opposite sites of the structure and the added segment crosses a significant part of the chromosome. The center of mass closure method reduces the risk that an added segment could change the knot type of individual chromosomes. However, those added segments form wide arcs, which can introduce additional links between chromosomes. As a result, one wouldn’t know whether the obtained link type actually indicates the linking between chromosomes or their closures. Link recognition in the above two methods is based only on one closure. Therefore, to increase the accuracy of link recognition, we claim that the most reliable closing procedure is to use a number of random closures, in particular when several chains are involved. The final result is then based on probability of occurring links. However, one should be aware that even in this approach there can appear some artificial linkings if two chromosomes are located close to each other.

**Figure 5:**
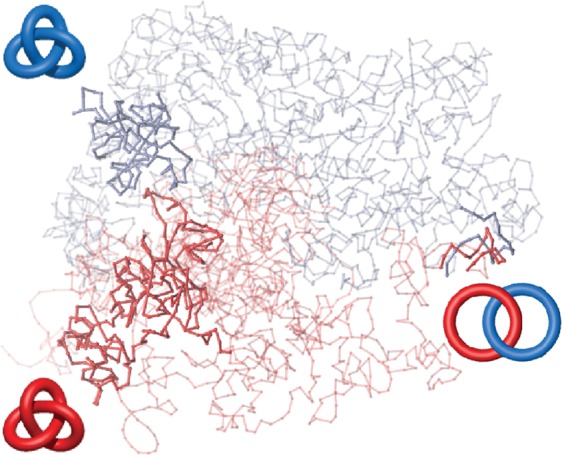
A pair of chromosomes *c* and *e* from the model 1 in the cell no. 7. Marked fragments of chromosomes represent knotted fragments of individual chains, and the link between the pair.

As an illustrative example of the link recognition method, we present a pair of chromosomes *c* and *e* from the model 1 in the cell no. 7. In this case each chain forms 3_1_ knot, *c* with the 90% probability, and *e* with 80% probability, both determined using the random closure method (see Fig. 5). The GLN value calculated for this pair is 1.15, indicating that both chromosomes are connected with the Hopf link (3_1_#3_1_#*Hopf*). Calculation of HOMFLY-PT polynomial for this pair of chromosomes predicts a link type 3_1_#3_1_#*Hopf* with probability 40%, however a few even more complicated topologies are also predicted with lower probabilities. At first sight, the probability of 40% for the most probable topology may seem to be rather small. If there was always the Hopf link between two chromosomes (as the GLN value indicates), topology of 3_1_#3_1_#*Hopf* would appear with probability around 72% (90%⋅80%). However, closing of each chain may not only influence the knot types of individual chains, but also the character of their linking. Let us assume that roughly one quarter of directions, in which we can connect chromosome ends, go through the other chromosome. These closures most likely alter the topology of the pair of chromosomes. In such case, the probability that the randomly chosen directions (i.e. closures) leave the link type unaffected, is around (1 − 1/4)⋅(1 − 1/4) = 9/16. This is because in order to preserve the link type, neither the closure of the first chromosome can cross the second chromosome, nor the closure of the second chromosome can cross the first chromosome, and both closing events are independent. This crude estimate leads to similar value as the probability predicted using random closure method (9/16⋅72% = 40.5%). In conclusion, the random closure method should lead to the most reliable results; note however, that this method is also the most time consuming one.

### Stable links

As mentioned above, we found 126 pairs of chromosomes with |*GLN*| > 0.7 in all eight cells, each represented by the structure from model no. 1. For each of these pairs we calculated |*GLN*| in all 9 other models from the original cell in order to observe link reproducibility. We found that 36 of them (29%) contained a stable link (with |*GLN*| > 0.7) in all 10 models for a given cell.

The presence of links predicted by our models can be challenged by studying conformational stability of the genome by (short) molecular dynamics simulations. Again, for each cell, we chose the model no. 1 as the starting structure. Then, we analyzed time evolution and topology conservation over time.

**Figure 6:**
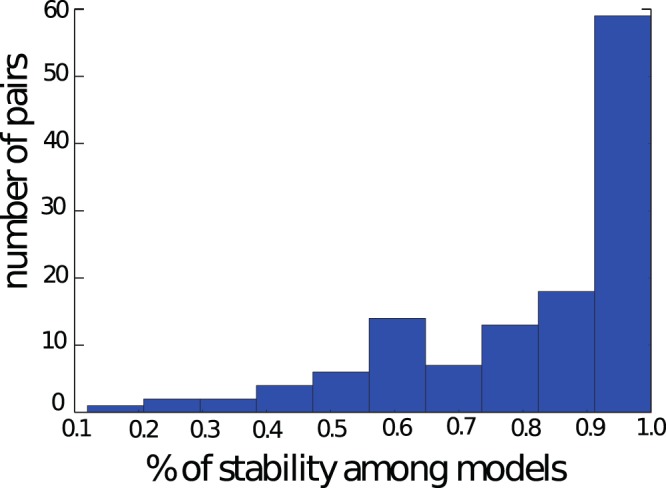
The number of chromosome pairs with |*GLN*| > 0.7 from all 8 cells as a function of trajectory fraction with conserved link. For each cell, model no. 1 was subject to relaxation.

First, we conducted the equilibrium dynamics simulations for temperatures within a range of 80–160 in reduced units and analyzed the RMSD of the simulated genome. The stability may strongly depend on the temperature value. Therefore, we chose the temperature equal to 120, in which the calculated RMSD of the structure is comparable with RMSD calculated for provided models and varies between cells (see Supplementary Material for details). When fluctuations of the processed structure lead to changes in the linking region, such a link is classified as unstable. If the link remains unchanged during molecular dynamics simulation, it is classified as a stable one. As a criterion for link stability during relaxation, we chose the condition that a link must be present in at least 90% of the recorded trajectory, while its GLN value changes by no more than ±0.2. As a result, from among 126 links with |*GLN*| > 0.7 we found 59 (47%) stable ones. The calculated distribution of links stability is presented in Fig. 6 (most of chromosome pairs remained linked with the starting GLN value within more than a half of a trajectory).

**Figure 7:**
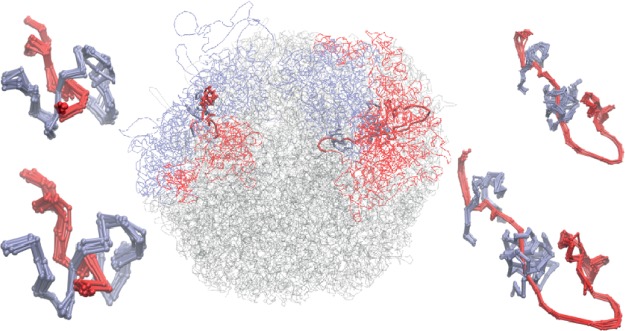
Middle: Structure of cell no. 5, model 1, with marked chromosome pairs *r*−*t* and *d*−*h*. Left top: superimposed 10 models of the linking region of the pair *r*−*t*. Left bottom: Superimposed snapshots from the relaxation for the pair *r*−*t*. Right top: superimposed 10 models of the linking region of the pair *d*−*h*. Right bottom: Superimposed snapshots from the relaxation for the pair *d*−*h*.

We identified 18 linked pairs of chromosomes as stable in both methods presented above. Their link type was determined by calculating HOMFLY-PT polynomials. Among them, we found only two relatively simple links, both from cell no. 5. The pair *d*−*k* was identified as 4_1_ # *Hopf* with 80% probability, and the pair *l*−*n* as 3_1_ # 3_1_ # *Hopf* with 40% probability. All other identified stable links are rather complex, with more than 8 crossings. Nine of them are identified in the cell no. 5. For two of them, pairs *d*−*h* (GLN ≈ −3.0) and *r*−*t* (GLN ≈ 1.2), the superimposed linking regions from the predicted models and from several trajectory snapshots are presented in Fig. 7. The pair *d*−*h* forms a highly complicated link, where the chromosome *h* wraps around the chromosome *d* three times, twice in a very close distance. These results are rather surprising, since we would expect to find rather simple stable links, as it was predicted for knots in a single chromosome^18,39,40^. Moreover, we found that the link (*d*−*h*) - classified as stable in two approaches - is also conserved between some cells. A few links which are stable in one of the methods are conserved between cells, e.g. the link between *f*−*g* is found in cells 4, 5 and 8, and the link between *h*−*m* is found in cells 4, 6 and 8.

**Figure 8:**
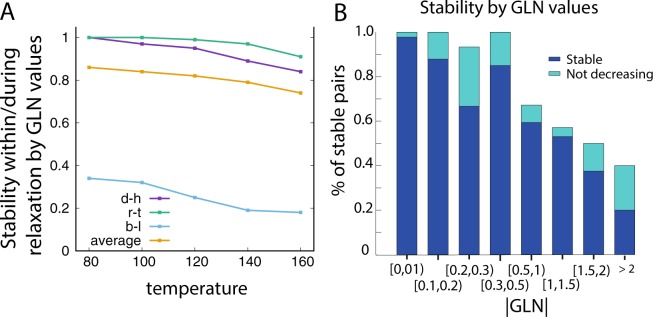
(**A**) Trajectory fraction with conserved link in relaxation simulations as a function of temperature (in reduced units). Three chromosome pairs belong to cell no. 5, model no. 1, and are analyzed as examples of stable and unstable links in section Results. Average link stability was calculated for all links with |*GLN*| > 0.7 in the cell no. 5, model no. 1. (**B**) Fraction of chromosome pairs with (blue) stable links and (cyan) links with not decreasing |*GLN*| value during relaxation, as a function of a given |*GLN*| range.

In Fig. 8A we present dependence of link stability on temperature for several cases, calculated from relaxation simulations for selected pairs. We stress that link stability prediction based on the molecular dynamics protocol may strongly depend on temperature, as well as on the applied stability criterion. Moreover, the link stability can also be defined in another way. Namely, one can think of a link as a stable one, when the |*GLN*| value does not decrease over the relaxation run, but it can increase with no restrictions. In such a way, a link is classified as stable when its complexity isn’t reduced. Comparison of this criterion with the criterion used throughout the article is shown in Fig. 8B.

### Unstable links

**Figure 9:**
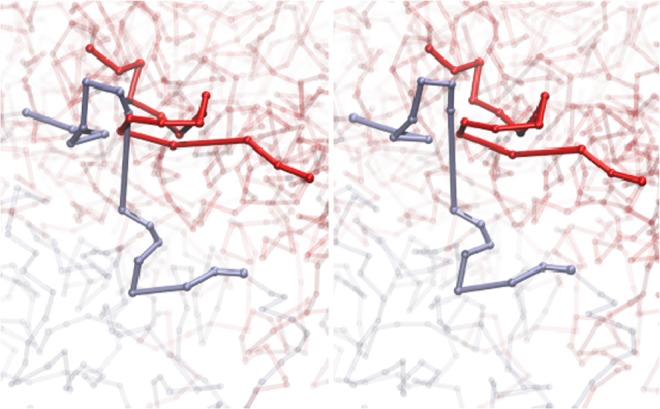
Snapshots from relaxation, pair of chromosomes *b* (red) and *l* (blue) from the cell no. 5, model no. 1; left: the pair is linked, right: the pair is unlinked. Denoted fragments of chromosomes represent the regions involved in link formation. These fragments were identified by studying shorter fragments with the GLN approach.

Some links are found to be unstable from model to model, or based on relaxation simulations. Such cases may arise when two chromatin fragments pass very close to each other. As an example of an unstable link, one can consider a pair of chromosomes *b* and *l* from the cell no. 5. Chromosome *b* contains 4_1_ knot with 45% probability, and chromosome *l* has 3_1_ knot with 75% probability based on random closure method for an individual chain. Calculated GLN was equal to 1.1, suggesting the Hopf link type for this pair. Calculation of HOMFLY-PT polynomial for this chromosome pair indicates the same result (link type 3_1_#4_1_#*Hopf*) as the most probable. During relaxation the link is present in 25% of simulation time, and no other linking is formed between these chromosomes (see Fig. 8 for stability dependence on temperature). Sample snapshots from the resulted trajectory are presented in Fig. 9. This link was also unstable based on the models analysis, where its presence was reported only in 3 out of 10 models. Recognition of unstable links together with chromatin fragments involved, allows determination of the sensitive regions and further investigation of their entanglement.

### Multiple links

**Figure 10:**
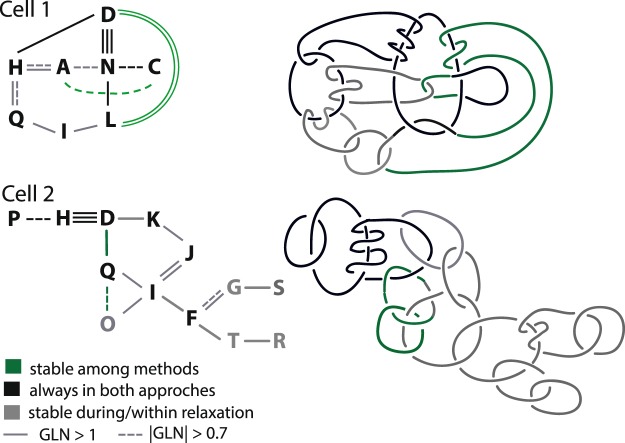
Examples of multiple stable links in cells no. 1 and 5. Top panel - a link composed of 8 chromosomes, with a chain of 4 chromosomes linked in stable way in both methods (in black). Bottom panel - a link composed of 13 chromosomes with two chains of three chromosomes linked in stable way in both methods. In left panels letters denote names of chains, right panels correspond to the left ones and visualize only the type of linking between chromosomes while neglect topology of single chromosomes (most of them are knotted). To make the scheme more readable chromosomes are presented here as closed circles.

Analysis of genomic 3D data provided in^21^ indicates that densely packed chromosomes can form much more complicated links. In the investigated nuclei of 8 cells we found several dozen links consisting of at least 3, and even up to 14 chromosomes each. From among them, the following four links composed of three and four chromosomes were stable in both models and relaxation: *c*−*n*−*d*−*h* (chromosome *c* linked with *n*, *n* linked with *d*, and *d* linked with *h*) from cell no. 1, *p*−*s*−*c* from cell no. 3, *d*−*h*−*p* and *j*−*i*−*q* both from cell no. 5. More complex links are schematically presented in Fig. 10. Detection procedure of such groups of linked chromosomes was based on the analysis of |*GLN*|, namely, we detected all these chromosomes which met the condition |*GLN*| > 0.7 with at least two other chromosomes.

## Discussion

In this work, based on the experimental data of the genome in the cell nucleus^21^, we have shown that chromosomes can be linked. We found numerous pairs of chromosomes that are entangled, some of them in a very complex way.

In fact, eighteen of identified pairs of chromosomes contain a stable link (in the sense that a similar high |*GLN*| value was repeated in all 10 models and a link type did not change during short molecular dynamics simulations). Furthermore, some of the links we found are preserved in different cells. Moreover, stable links are found to possess between 3 and 4 components, however those are not conserved between cells.

Almost all identified stable links have very complex topology (their diagrams have more than 9 crossings). These links, even though they are classified here as stable, should be treated with caution. Note, that in the case of a single chromosome, a simple topology of a trefoil knot (a knot with three crossings, 3_1_) was observed as a dominant one. Identified links are more complex than those found in proteins^29,31^.

Nevertheless, among stable links, we found also two pairs of chromosomes forming simple links: 4_1_#*Hopf* and 3_1_#3_1_#*Hopf*. These links are one strand passage distant from each other, which means that, by a single intersegmental strand passage, one of them can be transformed into another one^41,42^. Thus such links can be unlinked by one interchromosome strand passage, which can be performed by a topoisomerase^43^. This implies that such types of links do not block chromosome rearrangement, but can increase local stability as in the case of proteins^31,44^. Furthermore, local stability could be used as a spot to bind proteins.

The link analysis was supported with an alternative measure of mutual linkage of two loops - linking numbers. We detected links for which |*GLN*| ≥ 1 (*Hopf*, *Solomon*, etc.). However, there are some link types with *GLN* = 0 (e.g. Whitehead) which, when included, may even increase the number of already detected ones. We have shown that the linking number is also a useful tool for the further analysis of linked chains, since by studying shorter fragments of entangled chains one can specify precisely the linking region. One can then, by visual inspection, assess whether such a link may occur or if it is an effect of still low resolution of experimental data.

Known results suggest that DNA can be to some extent locally knotted, however, regulation of topoisomerase II activity and the fractal architecture of chromatin might be crucial to prevent a potentially massive and harmful self-entanglement of DNA molecules *in vivo*^20^. We are aware that our analysis was based on data with a resolution of 100 kb, which is not sufficient to unambiguously indicate the existence of links. However, identified links might suggest that chromosomes are entangled not only locally. How topoisomerase solves this topological problem remains an open question. On the other hand, methods presented here can be used as a quantitative assessment - descriptor - to distinguish quality of modeled data. Presented methods are freely available at the KnotGenome server http://knotgenom.cent.uw.edu.pl/^27^ and were inspired by methods developed to study entanglement in proteins, see servers KnotProt^26^, LinkProt^30^ and LassoProt^45^.

## Supplementary information

Supplementary File
