# Supplementary material for: Defining and detecting links in chromosomes
Source: Sci Rep. 2019 Aug 13;9:11753. doi: 10.1038/s41598-019-47999-4 (PMC6692345; doi:10.1038/s41598-019-47999-4)
Supplement: Supplementary file 1 — Supplementary File [file 41598_2019_47999_MOESM1_ESM.pdf]

Defining and detecting links in chromosomes

Supplementary Material

Szymon Niewieczerzal, Wanda Niemyska, and Joanna I. Sulkowska

Table 1: Number of links in all models from Stevens et al. [1] with condition $|GLN| > 0.7$

cells	models									
	1	2	3	4	5	6	7	8	9	10
1	20	18	20	21	19	18	20	19	19	19
2	7	7	6	8	8	6	7	7	8	5
3	25	24	27	26	31	33	27	28	26	28
4	12	12	11	15	12	14	12	12	11	12
5	21	17	19	15	18	21	18	20	19	15
6	15	14	12	10	11	11	12	15	14	13
7	13	13	9	12	9	7	10	9	7	8
8	14	12	11	15	13	14	13	13	14	11

Table 2: Number of links in all models from Stevens et al. [1] with condition $|GLN| > 1.5$

cells	models									
	1	2	3	4	5	6	7	8	9	10
1	7	6	8	7	4	6	8	5	6	5
2	0	0	0	1	1	0	0	2	1	1
3	10	9	11	10	12	14	11	12	12	10
4	2	1	1	1	1	1	1	1	1	2
5	3	2	3	3	3	4	3	3	3	2
6	0	1	1	1	0	1	1	2	0	0
7	1	2	0	1	1	0	1	1	2	1
8	4	4	4	4	3	2	4	4	4	4

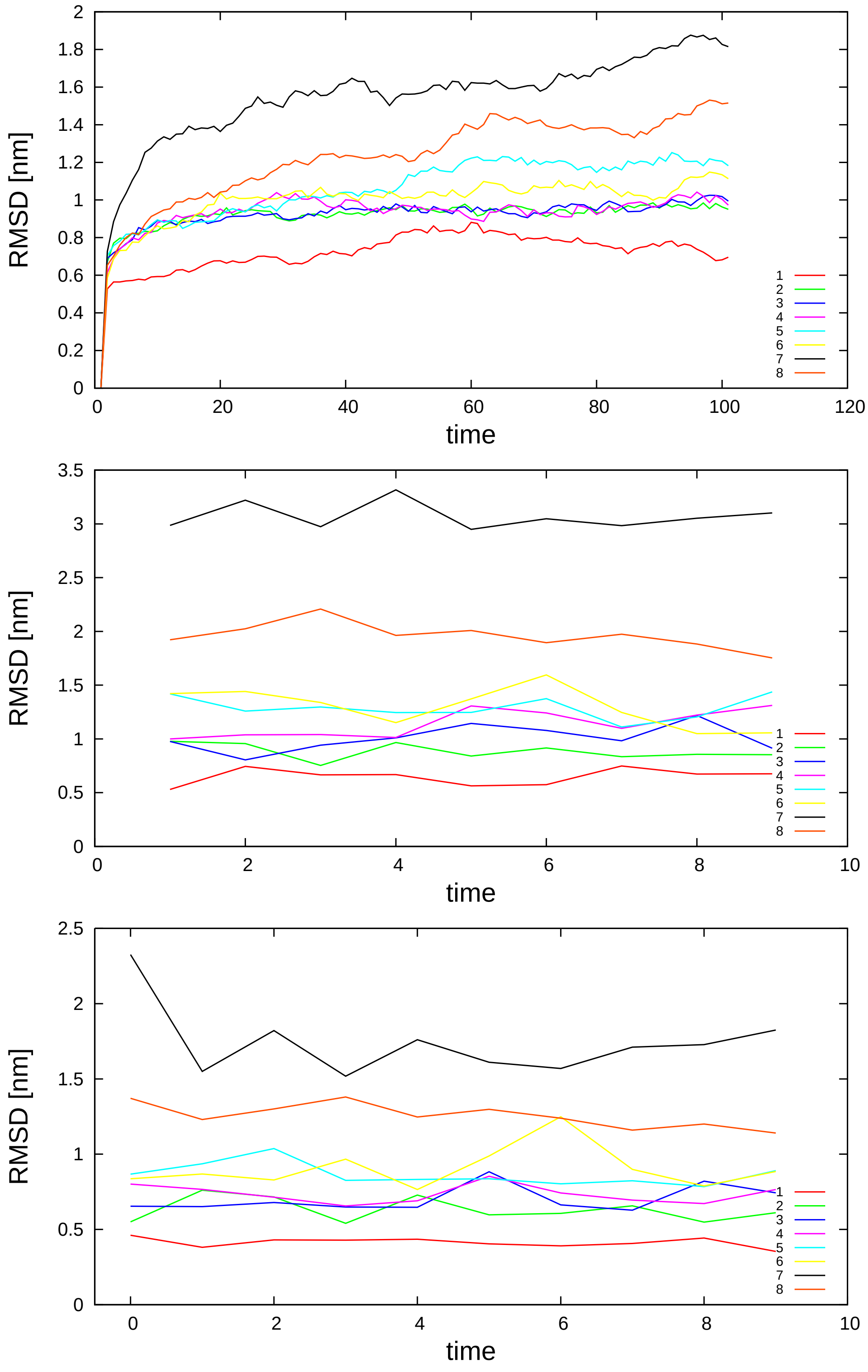

Figure 1: RMSD (top) from simulations, (middle) for models with model no. 1 as a reference structure, and (bottom) for models compared with the average structure.

References

[1] Stevens, T. J. *et al.* 3d structures of individual mammalian genomes studied by single-cell hi-c. *Nature* **544**, 59 (2017).
